# Identification of a New Pentafluorosulfanyl-Substituted Chalcone with Activity Against Hepatoma and Human Parasites

**DOI:** 10.3390/ph18010050

**Published:** 2025-01-03

**Authors:** Alessandra Viperino, Michael Höpfner, Nicole Edel, Ibrahim S. Al Nasr, Waleed S. Koko, Tariq A. Khan, Imen Ben Abdelmalek, Rainer Schobert, Bernhard Biersack, Bianca Nitzsche

**Affiliations:** 1 Institute of Physiology, Charité-Universitätsmedizin Berlin, Corporate Member of the Freie Universität Berlin, Humboldt-Universität zu Berlin and Berlin Institute of Health, Charitéplatz 1, 10117 Berlin, Germany; alessandra.viperino@charite.de (A.V.); michael.hoepfner@charite.de (M.H.); nicole-edel@t-online.de (N.E.); 2 Department of Biology, College of Science, Qassim University, Qassim 51452, Saudi Arabia; insar@qu.edu.sa (I.S.A.N.); wasyko2002@yahoo.com (W.S.K.); mm.abdulmalek@qu.edu.sa (I.B.A.); 3 Department of Basic Health Sciences, College of Applied Medical Sciences, Qassim University, Ar Rass 51921, Saudi Arabia; sirtariqayub@gmail.com; 4 Organic Chemistry Laboratory, University Bayreuth, Universitätsstrasse 30, 95440 Bayreuth, Germany; rainer.schobert@uni-bayreuth.de

**Keywords:** chalcone, anticancer agent, antiparasitic agent, liver cancer, leishmaniasis, toxoplasmosis, pentafluorosulfanyl group, anthracene, ferrocene, Hsp90

## Abstract

Background/Objectives: New drugs are required for the treatment of liver cancers and protozoal parasite infections. Analogs of the known anticancer active and antileishmanial 2′,4′,6′-trimethoxychalcone SU086 were prepared and investigated. Methods: The chalcones were prepared according to the Claisen–Schmidt condensation protocol and analyzed. They were tested for activity against two liver cancer cell lines (HepG2 and HuH-7) and protozoal parasites (*Toxoplasma gondii* and *Leishmania major*). Unspecific toxicity and expression of Hsp90 and Hsp70 upon treatment were analyzed in liver cancer cells. Results: A new chalcone, 2′,4′,6′-trimethoxy-3-pentafluorosulfanylchalcone (246TMP-3SF5), with a pentafluorosulfanyl (SF_5_) substituent showed pronounced activities against liver cancer cells and *T. gondii* parasites which were superior to the activities of the parent chalcone SU086 in these models. In contrast, SU086 and its anthracene analog 2′,4′,6′-trimethoxy-9-anthracenylchalcone (246TMP-Anth) were most active against *L. major* promastigotes. The new SF_5_-substituted chalcone behaved like the known Hsp90 inhibitor 17-AAG and upregulated Hsp70 expression in liver cancer cells. Conclusions: The SF_5_-substituted SU086 analog has potential to become a new drug for the therapy of hepatoma and toxoplasmosis.

## 1. Introduction

**Figure 1:**
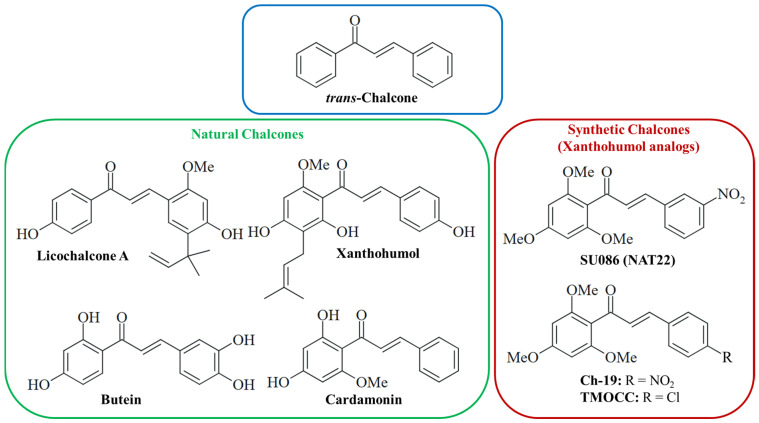
Structures of the core compound *trans*-chalcone and of notable natural or synthetic chalcone derivatives with known anticancer activities.

While synthetic chalcones (1,3-diarylprop-2-en-1-ones, with *trans*-chalcone as the basic/core chalcone compound, Figure 1) can be prepared by simple condensation reactions such as the Claisen–Schmidt reaction, natural chalcones are metabolites of various plants and essential intermediates for the biosynthesis of (iso-)flavonoids. Both natural and synthetic chalcones are a highly prolific class of bioactive compounds, and their medicinal properties, which also include pronounced anticancer and anti-infective effects, were thoroughly studied [1,2,3]. In terms of anticancer active chalcones, several inhibitors of tubulin polymerization, ABC transporters (e.g., BCRP/ABCG2), STAT3 and NF-κB, were disclosed [4,5]. Trimethoxyphenyl-based chalcones are of particular interest as inhibitors of tubulin polymerization, TrxR, and Hsp90 [6,7,8,9]. The 2′,4′,6′-trimethoxychalcone SU086 (aka NAT22) also showed high activity against human pathogenic *Leishmania* parasites (Figure 1) [10]. Thus, SU086 has the potential for the treatment of various life-threatening human diseases.

Hepatocellular carcinoma (HCC) is the most abundant primary liver cancer worldwide and is characterized by a high lethality [11]. The main risk factors for the development of HCC are hepatitis infection, excessive alcohol consumption, and non-alcoholic fatty liver disease [12]. Patients suffering from advanced HCC have an especially poor prognosis. Current therapy options of advanced HCC include some small-molecule multikinase inhibitors (sorafenib, lenvatinib, regorafenib and cabozantinib), the VEGFR inhibitor antibody bevacizumab, and immune checkpoint inhibitors (atezolizumab, nivolumab, pembrolizumab), as well as combinations thereof [13]. However, the outcome of these therapies is limited because of the emergence of kinase inhibitor resistance and immune evasion processes [14,15]. Thus, new and more effective drug candidates for the therapy of HCC are required. Several natural chalcones were reported to have potent anti-HCC activities including apoptosis induction (xanthohumol, butein and cardamonin), interference with vital oncogenic signaling pathways (Notch signaling by xanthohumol, Akt signaling by isoliquiritigenin) and protein kinases (EGFR and aurora B kinase by butein), and inhibition of migration and invasion (licochalcone A and xanthohumol, Figure 1) [16,17,18,19,20,21,22]. Notably, licochalcone A in combination with sorafenib exhibited synergy effects in terms of HCC metastasis inhibition in vitro and in vivo [23]. Several other synthetic chalcones with considerable activity against HCC were described. The development of SU086, which targets Hsp90 and TrxR, was inspired by the hop ingredient xanthohumol and SU086 was reportedly active against hepatoma cells [8,9]. The 4-nitro analog of SU086, Ch-19, induced ROS formation and apoptosis in esophageal cancer cells (Figure 1) [24]. Another analog with a chloro-substituent, dubbed TMOCC, led to apoptosis and DNA strand breaks in HCC cells, and inhibited AKT/FOXO3a and Ras-MAPK signaling (Figure 1) [25]. Further synthetic chalcones induced apoptosis (dimethoxy- or triethoxyphenyl-based chalcones, nitrophenyl chalcones) or autophagy (*trans*-chalcone), inhibited signaling pathways (AKT by nitrophenyl chalcones, STAT3/STAT5 by thiopyrimidine-chalcone hybrids and Wnt signaling by *trans*-chalcone), and enhanced ROS formation (dimethoxyphenyl-based chalcones and *trans*-chalcone) in HCC models [26,27,28,29,30]. Coumarin-chalcone hybrids with antibacterial and anti-HCC activity were also reported [31].

Exposure to anticancer drugs means substantial stress for the affected cancer cell. However, a high tolerance to stress factors was observed in drug-resistant tumor cells, which was correlated with an increased expression of heat shock proteins (Hsps), aka chaperones, responsible for the stabilization of client proteins such as p53 and protein kinases (Her2, BRAF, AKT and CDK4) [32]. The overexpression of Hsps such as Hsp27, Hsp70, Hsp90, Grp78 and Grp94 was associated with hepatocarcinogenesis, while increased levels of Hsp90, Grp78 and Grp94 were connected with HCC metastasis and vascular invasion [33]. The deregulation of Hsp activities in hepatocellular carcinoma also promotes tumor cell proliferation and thus the inhibition of crucial Hsps is an attractive strategy to treat HCC [34,35]. In detail, Hsp90 prevents the misfolding of oncoproteins which foster vital signaling pathways ultimately leading to tumorigenicity and drug resistance [36]. Sorafenib resistance in HCC was overcome by treatment with the Hsp90 inhibitor 17-AAG [37]. Hsp70 and the co-chaperone Hsp40 have also shown oncogenic effects in HCC which might be tackled by Hsp70 and/or Hsp40 modulators [34,38].

Infections of protozoal parasites are a considerable health problem worldwide. Leishmaniasis is classified as a neglected tropical disease (NTD) and manifests in three different forms, cutaneous leishmaniasis (CL), mucocutaneous leishmaniasis (MCL), and visceral leishmaniasis (VL) [39]. CL is the most common disease form and is characterized by severe and disfiguring skin lesions. The kinetoplastid parasites *L. major* and *L. tropica* are the causative agents of CL, and *L. major* is responsible for 50% of the leishmaniasis cases detected in central Saudi Arabia (Qassim Province) [40]. The wide distribution of desert rodents (reservoirs) and sand flies (vectors) in Arabia explains the endemic character of CL in this region [41,42]. New treatments of leishmaniasis are required since currently applied drugs are toxic (antimonials) or not available (amphotericin B) in certain regions [43]. Several chalcones including licochalcone A, SU086, etc., have shown promising activities against various *Leishmania* species and might become suitable drug candidates for the therapy of leishmaniasis [10,44,45,46,47,48,49].

Malaria and toxoplasmosis are wide-spread infectious diseases caused by apicomplexan parasites (*Plasmodium* species and *Toxoplasma gondii*). While many efforts are currently undertaken to develop new antimalarial drugs, in particular, against resistant malaria forms, antitoxoplasmal drug development is still lacking a broader interest [50,51]. Toxoplasmosis is a zoonosis mainly transmitted by cats [52,53]. It is associated with life-threatening complications (e.g., development of brain cysts) in immunocompromised persons and thus an efficient way to manage the infection in vulnerable people is required. Currently, the arsenal of available antitoxoplasmal drugs includes sulfadiazine (DHPS inhibitor), pyrimethamine and trimethoprim (DHFR inhibitors) and atovaquone (a redox-active naphthoquinone) [54]. The identification of more suitable hosts and/or parasite targets is required for the design of new promising antitoxoplasmal drug candidates [55]. Notably, *T. gondii* Hsp90 and Hsp70 were found to be crucial for the pathogenic properties of the parasite upon host infection [56]. Certain chalcone derivatives such as licochalcone A, as well as synthetic chalcone and bichalcone compounds have already shown considerable activity against *T. gondii* [57,58,59].

In this work, we studied the anticancer and antiparasitic activities of a new SU086 chalcone derivative with a pentafluorosulfanyl group (SF_5_, aka “super trifluoromethyl group”) instead of the nitro group [60]. In addition, further known SU086 analogs with anthracene and ferrocene moieties were prepared and tested for anti-hepatoma and antiparasitic effects for comparison purposes. Various ferrocene derivatives have shown distinct biological activities including anticancer and antiparasitic properties, while anthracenes are potent DNA binders [61,62].

## 2. Results

### 2.1. Chalcone Test Compounds

**Figure 2:**
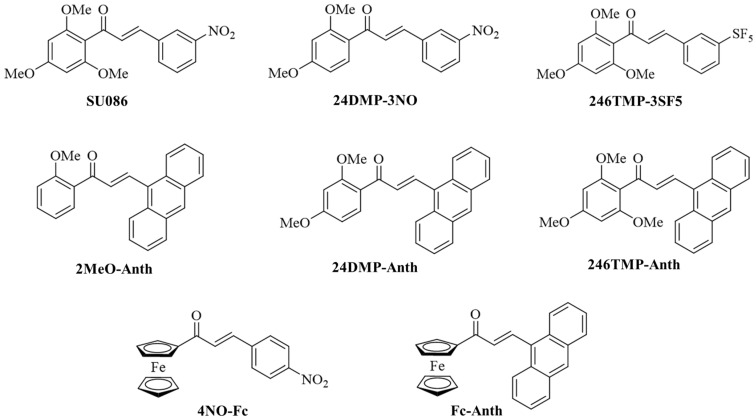
Structures of chalcones used in this study for anti-hepatoma and antiparasitic testing.

In addition to SU086, five known chalcones and two new derivatives 2′,4′,6′-trimethoxy-3-pentafluorosulfanylchalcone (246TMP-3SF5) and 2′,4′,6′-trimethoxy-9-anthracenylchalcone (246TMP-Anth) were synthesized and evaluated in this study (Figure 2). The chalcone test compounds were prepared via the straightforward Claisen–Schmidt condensation reaction in ethanol under basic conditions (aqueous NaOH) at room temperature (Scheme S1). The new chalcones 246TMP-3SF5 and 246TMP-Anth were obtained as yellow solids and analyzed by NMR (nuclear magnetic resonance) spectroscopy and HRMS (high-resolution mass spectrometry, Figures S1–S5). The observed ^1^H NMR coupling constant (J) of 16.0 Hz or 16.3 Hz, respectively, for the olefin protons indicates the *trans*-configuration of the new chalcones. The ^1^H NMR spectra of the other known chalcones also provides evidence of *trans*-configurations (J = 15.9–16.1 Hz). HRMS analysis confirmed the molecular mass of both new chalcones plus one proton.

### 2.2. Activity Against Liver Cancer Cells

The antiproliferative activities of the chalcones described in Figure 2 were tested against HepG2 and HuH-7 HCC cells (Table 1). In addition to the parent chalcone SU086, the Hsp90 inhibitor 17-AAG (tanespimycin) and the multi-kinase inhibitor sorafenib (currently used to treat advanced HCC) were applied as positive control drugs. Among the tested chalcones, only the combination of a 2,4,6-trimethoxyphenyl moiety with a 3-SF_5_-substituted phenyl group (246TMP-3SF5), and that of ferrocene with nitrophenyl (4NO-Fc) appeared to be favorable in terms of anticancer activity against HCC. The chalcones with 3-nitrophenyl or anthracene moieties were inactive at concentrations at or below 10 µM, including parent chalcone SU086. The most active chalcone 246TMP-3SF5 was distinctly more active than sorafenib, while 4NO-Fc showed activities similar to sorafenib. 246TMP-3SF5 and 17-AAG exhibited comparable activities against HepG2 cells, while 17-AAG was more active against HuH-7 cells.

**Figure 3:**
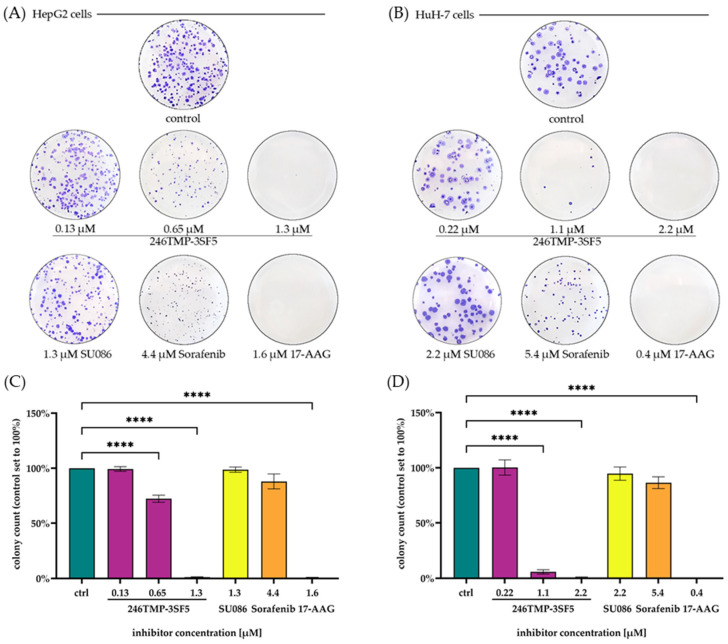
Colony formation after 14 days of growth under chalcone 246TMP-3SF5 treatment or given inhibitor at the indicated concentrations in HepG2 (**A**) and HuH-7 cells (**B**). Colony count was assessed using ImageJ and quantified for HepG2 (**C**) and HuH-7 cells (**D**). Results are shown as means ± SEM of *n* = 4 independent experiments. **** *p* ≤ 0.0001; one-way ANOVA Dunnett’s post-hoc test.

246TMP-3SF5 showed the greatest antiproliferative effects in a short-term analysis of antiproliferative efficacy amongst the synthesized chalcones and was thus selected for further investigations on its therapeutic effects against HCC cells. To assess the long-term effects of 246TMP-3SF5 on proliferation as well as the clonogenicity of HCC cell lines colony formation assays were performed (Figure 3). Both HepG2 and HuH-7 cells showed a dose-dependent decrease in colonies formed when treated with 246TMP-3SF5. Matching the results of the previous antiproliferative assessment SU086 shows no long-term antiproliferative effects, while sorafenib decreased the size of the colonies formed; however, the colony count did not differ much from the untreated controls. 17-AAG and 246TMP-3SF5 inhibited colony formation significantly. At concentrations similar to its IC_50_ value, 246TMP-3SF5 decreased the colonies formed after two weeks of growth down to almost none, in both cell lines respectively, exhibiting pronounced short- and long-term antiproliferative effects and simultaneously reducing the clonogenicity in the HCC cell lines.

**Figure 4:**
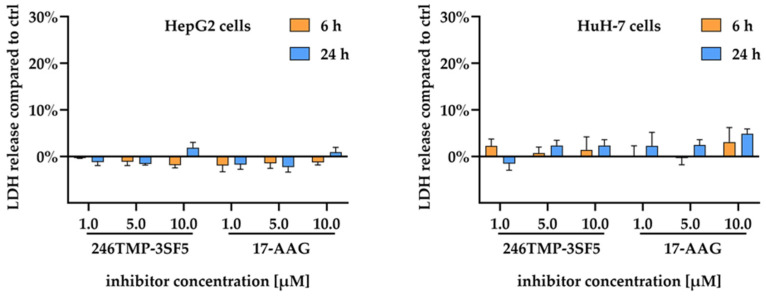
LDH assay with chalcone 246TMP-3SF5 and comparison with the Hsp90 inhibitor 17-AAG after 6 and 24 h of treatment at the indicated concentrations in HepG2 and HuH-7 cells. Data are given as changes in percentage compared to LDH release of untreated controls. Results are shown as means ± SEM of *n* = 3 independent experiments.

Non-specific toxicity of 246TMP-3SF5 in HCC cells was evaluated by the lactate dehydrogenase (LDH) assay which detects unspecific damage and leaks of the cancer cell membrane via the degree of LDH release (Figure 4). It was shown that the chalcone 246TMP-3SF5 does not significantly increase LDH release time- or dose-dependently as a means of non-specific toxicity at the indicated concentrations after 6 and 24 h which excludes this mechanism of action in liver cancer cells for this compound.

**Figure 5:**
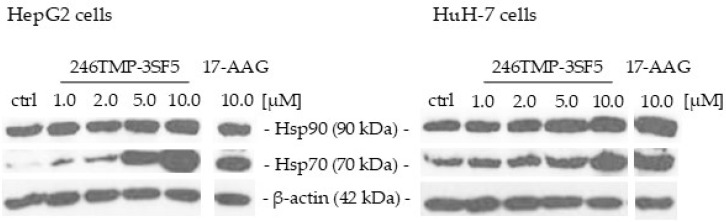
Western Blot images showing Hsp90, Hsp70 and β-actin expression levels in HCC cell lines upon treatment with 246TMP-3SF5 and comparison compound 17-AAG for 24 h at indicated concentrations. Representative Western blot images of *n* = 3 independent experiments are shown.

As 246TMP-3SF5 is derived from a Hsp90 inhibitor, we further assessed changes in the protein expression levels of the targeted key heat shock proteins Hsp90 and Hsp70 in Western blot analysis (Figure 5). 246TMP-3SF5 treatment resulted in almost no changes in the expression of Hsp90. However, Hsp70 expression drastically increased upon treatment with 246TMP-3SF5 in a dose-dependent manner in both HCC cell lines. While the strongest induction of Hsp70 was observed after treatment with 10 µM 246TMP-3SF5 in both cell lines, 17-AAG showed a similar induction in Hsp70 expression. The level of Hsp90 expression remained almost unchanged.

### 2.3. Antiparasitic Activity

The antiparasitic activities of the chalcones indicated in Figure 2 were initially tested using *T. gondii* parasites, and the results were compared with the toxic effects on non-malignant Vero kidney cells as host cells (Table 2). Atovaquone (ATO) was applied as a positive control drug [63]. 246TMP-3SF5 was the most active chalcone with an excellent IC_50_ value of 0.42 µM, but less active than the approved drug atovaquone. Yet, it was distinctly more active than SU086 (IC_50_ = 3.8 µM) and the other chalcones. Interestingly, 24DMP-3NO (IC_50_ = 2.2 µM) was slightly more active against *T. gondii* than SU086. Albeit being moderately active against hepatoma, chalcone 4NO-Fc turned out to be the least active chalcone against *T. gondii* (IC_50_ = 18.8 µM). Moderate toxicities in Vero cells and associated selectivities (SI = 2.4–3.4) were observed for the active chalcone derivatives.

The chalcones were also tested for activity against *L. major* parasites (promastigote and amastigote forms) using amphotericin B (AmB) as a positive control drug (Table 3) [63]. The obtained results were compared with compound toxicities in macrophages (host cells). The 3-nitrophenyl derivatives SU086 and 24DMP-3NO, as well as the new anthracene analog 246TMP-Anth, showed high activity (IC_50_ < 1 µM) against *L. major* promastigotes. Activities against amastigotes were distinctly lower (only SU086 exhibited an IC_50_ value of 3.5 µM below 10 µM), as were their toxicities to macrophages. Thus, the active chalcones showed considerable SI values (21.7–29.3) in terms of activity against promastigotes. SU086 was the most active chalcone against *L. major* promastigotes and amastigotes. In contrast, 246TMP-3SF5, which was the most active chalcone in the assays with liver cancer cells and *T. gondii* parasites, was the least active one in the experiments with *L. major* promastigotes.

## 3. Discussion

The evaluation of a series of chalcones, which are structurally related to the known Hsp90 inhibitor and antileishmanial compound SU086, in various liver cancer and protozoal parasite models led to remarkable and, in parts, unexpected results. In particular, the observed anticancer effects of 246TMP-3SF5, the new chalcone derivative with a pentafluorosulfanyl substituent instead of the nitro group characteristic of SU086, on liver cancer cells are noteworthy. In two HCC cell lines, 246TMP-3SF5 revealed pronounced antiproliferative effects with low micromolecular IC_50_ values of 1.3 ± 0.2 µM and 2.2 ± 0.3 µM after 48 h for HepG2 and HuH-7 cells, respectively. In contrast, SU086 showed no antiproliferative effects in HCC cell lines at concentrations below 10 µM. Compared with sorafenib, a currently approved drug for the treatment of advanced stages of HCC, 246TMP-3SF5 displayed greater antiproliferative effects, both in short- and long-term antiproliferative analysis. While 17-AAG, a known Hsp90 inhibitor, and 246TMP-3SF5 exhibited similar antiproliferative efficacy in HepG2 cells with comparable IC_50_ values, 17-AAG reached a lower IC_50_ value in HuH-7 cells. However, the long-term analysis of antiproliferative effects revealed a similar potency of 246TMP-3SF5 and 17-AAG with close to no colonies formed after growth for 14 days in both HepG2 and HuH-7 cells. While 17-AAG has already been evaluated in preclinical studies demonstrating the great potential of the drug, clinical evaluations of its applicability for the treatment of different cancer entities have been limited by the occurrence of hepatotoxic side effects [64]. In this context, at least the preclinical evaluation of 246TMP-3SF5 excluded unspecific toxicity as the prominent mode of action in HCC cell lines. Therefore, 246TMP-3SF5 has the potential to kill cancer cells selectively with its specific mechanisms of cell death to be elucidated in future studies.

Analyzing the 246TMP-3SF5-induced protein expression of Hsp90 and Hsp70 in HCC cells yielded a dose-dependent increase in Hsp70 expression, an effect that was also observed for 17-AAG-treated cells. The elevated expression of Hsp70 in HCC cell lines upon treatment with Hsp90 inhibitors has been shown before but did not alleviate the anti-tumor effects of the inhibitors [65]. The cellular localization of the produced Hsp70 proteins might play a role because it was shown that an increased Hsp70 presence in cancer cell membranes can mediate anticancer immunity, while cytosolic Hsp70 is more cell-protective [66]. In addition, the Hsp70 suppressing pan-CDK inhibitor AT7519 can be beneficial in combination with Hsp90 inhibitors that upregulate Hsp70 expression in cancers (usually upregulated by inhibitors targeting the NTD/N-terminal domain ATP binding site of Hsp90, e.g., geldanamycin and its derivatives such as 17-AAG) [67]. In contrast to Hsp70, the expression of Hsp90 proteins remained relatively unaltered in cells treated with 246TMP-3SF5. However, further studies on the impact of this novel chalcone on the activity of Hsp90 and its client proteins are required to decipher its Hsp90-inhibitory mode in depth. Nevertheless, the induction of the expression of Hsp70, but not of Hsp90, in analogy to the effects of the known NTD-targeting Hsp90 inhibitor 17-AAG, can already be a hint at Hsp90 inhibition via the NTD by the chalcone 246TMP-3SF5.

Meanwhile, the SF_5_ group was also successfully applied in the design of other an-ticancer agents such as inhibitors of nuclear receptors (androgen receptor and progesterone receptor), and HDACs, as well as in pro-apoptotic curcumin derivatives [68,69,70,71,72]. The ferrocene derivative 4NO-Fc was also active against hepatoma cells while its anthracene analog was inactive, as were all anthracenes in this study. The activity of 4NO-Fc against HCC cells appears to be higher when compared with previously published ferrocene derivatives [73,74].

A clear difference between SU086 and 246TMP-3SF5 was also found in terms of their antiparasitic activity. In contrast to SU086, the new chalcone 246TMP-3SF5 was very active against *T. gondii*. However, it was much less antileishmanial in *L. major* promastigotes than SU086. The reasons behind this difference in antiparasitic activity by changing a single substituent remain obscure. It is conceivable that cell death mechanisms play a considerable role in the antitoxoplasmal activity of 246TMP-3SF5, since treatment of *T. gondii* with the drug dinitolmide was shown to induce an upregulation of apoptosis-related genes followed by parasite cell death [75]. The potent antitoxoplasmal activity of the SF5-substituted chalcone 246TMP-3SF5 adds well to the reported antimalarial activities of 8-SF_5_-mefloquine and SF_5_-modified *N*-heterocyclic *Pf*DHODH inhibitors which exhibited improved pharmacokinetics and in vivo activity in mice infected with *P. falciparum* or *P. berghei* strains [76,77,78]. Several SF_5_-substituted inhibitors of human DHODH were also described [79].

In contrast to the SF_5_-analog, the anthracene-based chalcone 246TMP-Anth showed sub-micromolar activity against *L. major* promastigotes, which were generally quite sensitive to anthracene and 3-nitrophenyl derivatives. Known mechanisms of action of anthracenes and nitroarenes (e.g., ROS formation and DNA targeting) might play a special role in the increased toxicity of these chalcones to promastigotes [61,62]. Since naphthalene-based chalcones have recently shown promising anticancer and antimicrobial activities, a replacement of the anthracene moiety by a naphthalene fragment might be considered in future studies [80].

Thus, two new and structurally simple SU086-derived chalcones (246TMP-3SF5 and 246TMP-Anth) with pronounced activities against HCC and/or parasite cells were identified. Their synthesis is straightforward and cost-effective, which can become relevant at later stages of their development as drugs. For instance, the development of the Hsp90 inhibitor 17-AAG as a new anticancer drug was halted at advanced clinical stages because of economic reasons [81]. Further investigations of the mechanisms of action of 246TMP-3SF5 and 246TMP-Anth will provide more insights into the anticancer and antiparasitic potential of these compounds (e.g., cancer entities other than liver cancer and effects on other protozoal parasites) and their prospects as new treatment options.

## 4. Materials and Methods

### 4.1. Chemistry

Starting compounds were purchased from the common providers. 9-Formyl anthracene was prepared from anthracene under Vilsmeiser reaction conditions [82]. The known chalcones SU086, 24DMP-3NO, 2MeO-Anth, 24DMP-Anth, 4NO-Fc and Fc-Anth were prepared according to literature procedures [8,83,84,85,86]. The devices and equipment used for the analysis of the new chalcones were mentioned previously [63,87].

#### 4.1.1. 2′,4′,6′-Trimethoxy-3-pentafluorosulfanylchalcone (246TMP-3SF5)

2,4,6-Trimethoxyacetophenone (210 mg, 1.0 mmol) and 3-(pentafluorothio)benzaldehyde (232 mg, 1.0 mmol) were dissolved in EtOH (10 mL) and aqueous NaOH (40%, 1 mL) was added. The reaction mixture was stirred at room temperature for 24 h. The formed precipitate was collected, washed with EtOH and dried. Yield: 220 mg (0.52 mmol, 52%); yellow solid of mp 123 °C; *υ*_max_(ATR)/cm^−1^ 3002, 2978, 2939, 2845, 1651, 1588, 1466, 1415, 1339, 1296, 1267, 1228, 1206, 1159, 1125, 1185, 1084, 1046, 1031, 976, 953, 923, 843, 812, 799, 681; ^1^H NMR (300 MHz, CDCl_3_) *δ* 3.77 (6 H, s), 3.85 (3 H, s), 6.15 (2 H, s), 6.98 (1 H, d, J = 16.0 Hz), 7.35 (1 H, d, J = 16.0 Hz), 7.4-7.5 (1 H, m), 7.6–7.8 (2 H, m), 7.83 (1 H, s); ^13^C NMR (75.5 MHz, CDCl_3_) *δ* 55.5, 55.9, 90.8, 111.4, 125.6, 126.9–127.1 (m), 129.2, 130.7, 131.0, 136.2, 141.1, 154.2–154.6 (m), 159.1, 162.8, 193.3; HRMS for C_18_H_18_O_4_F_5_S [M^+^ + H] calcd. 425.08405, found 425.08222.

#### 4.1.2. 2′,4′,6′-Trimethoxy-9-anthracenylchalcone (246TMP-Anth)

Analogously, 246TMP-Anth was obtained as a yellow solid from 2,4,6-trimethoxyacetophenone (210 mg, 1.0 mmol) and 9-formyl anthracene (206 mg, 1.0 mmol). Yield: 243 mg (0.61 mmol, 61%); ^1^H NMR (300 MHz, CDCl_3_) *δ* 3.85 (3 H, s), 3.88 (6 H, s), 6.20 (2 H, s), 6.92 (1 H, d, J = 16.3 Hz), 7.4–7.5 (4 H, m), 7.9–8.0 (2 H, m), 8.2–8.4 (3 H, m), 8.42 (1 H, s); HRMS for C_26_H_23_O_4_ [M^+^ + H] calcd. 399.15909, found 399.15798.

### 4.2. Biological Evaluations

#### 4.2.1. Compounds

Stock solutions (20 mM) of the compounds were prepared using dimethyl sulfoxide (DMSO, Sigma-Aldrich, Darmstadt, Germany) and stored at −20 °C. Work solutions (1 mM) were freshly prepared for the experiments by diluting the stock solution with medium and stored at 4 °C for up to two weeks.

The Hsp90 inhibitor 17-AAG was purchased from AbMole BioScience (Houston, TX, USA, Cat. No. M2320) and the multikinase inhibitor sorafenib was purchased from Targetmol (Boston, MA, USA, Cat. No T0093L).

#### 4.2.2. Cell Culture

Human hepatocellular carcinoma cell lines HepG2 (ATCC number: HB-8065) and HuH-7 (JCRB number: JCRB0403) were cultured at Roswell Park Memorial Institute (RPMI) 1640 Medium (Gibco, Thermo Fisher Scientific, Waltham, MA, USA) supplemented with 10% fetal bovine serum, 2 mM L-glutamine (Corning, Glendale, CA, USA), 100 U/mL penicillin and 100 µg/mL streptomycin (Bio&SELL, Feucht, Germany) at 37 °C, 5% CO_2_ in a humidified atmosphere in an incubator.

#### 4.2.3. Antiproliferative Activity Assessment

Treatment-induced antiproliferative effects measured as a decrease in cell number were assessed by crystal violet (N-hexamethylpararosaniline from Sigma-Aldrich, Darmstadt, Germany) staining as described before [71,88]. In brief, HepG2 and HuH-7 cells were seeded in 96-well plates and allowed to adhere to the bottom of the wells and grow for 72 h in an incubator (37 °C, 5% CO_2_, humidified atmosphere). Afterwards, the cells were treated with rising concentrations of the chalcones, sorafenib and 17-AAG. After 24 h, 48 h and 72 h, respectively, the cells were fixed using 1% glutaraldehyde and stained for 30 min with 0.1% crystal violet. The excess unbound dye was removed by rinsing with water for 30 min. Cells were then lysed by incubation with 0.2% Triton X-100 (Sigma-Aldrich, Darmstadt, Germany) overnight dissolving the bound dye. The extinction of crystal violet was measured at 570 nm using ELISA readers (Dynex Technologies, Denkendorf, Germany; GloMax^®^ Discover Microplate Reader Promega, Madison, WI, USA). IC_50_ values are given as means ± SD of a minimum of three independent experiments performed in triplicates and more.

#### 4.2.4. Colony Formation Assay

Long-term effects of drug treatment on proliferation and clonogenicity were assessed by colony formation assays. HCC cell lines were seeded at 1000 cells/well (HepG2) and 200 cells/well (HuH-7) in 6-well plates and allowed to adhere overnight. Thereafter, the cells were treated with sorafenib, 17-AAG and 246TMP-3SF5. Untreated cells served as controls. After 14 d, the cells were washed with PBS, fixed with 4% formaldehyde for 1 h and stained with 0.5% crystal violet for 3 min. Excess crystal violet dye was removed by gently rinsing with water. Representative images were taken and stained colonies were counted. A colony was defined as a cell aggregate of 50 or more cells. *n* = 4 independent experiments were performed and significance was tested by one-way ANOVA assays.

#### 4.2.5. Lactate Dehydrogenase Assay

To exclude unspecific compound-induced cytotoxicity as the predominant mode of action a lactate dehydrogenase (LDH) assay was performed using the Cytotoxicity Detection Kit^PLUS^ LDH (Roche Diagnostics GmbH, Mannheim, Germany, Cat. No. 4744934001) as described earlier [88]. In short, HCC cells were seeded in 96-well plates at 8 × 10^3^ cells/well (HepG2) and 10 × 10^3^ cells/well (HuH-7). Following incubation overnight, the cells were treated with 246TMP-3SF5 and 17-AAG at final concentrations of 1 µM, 5 µM and 10 µM, respectively, for 6 h and 24 h. The formation of the red-brownish formazan dye, which is formed proportionally to the LDH enzyme activity in the sample, was measured at 490/600 nm using the GloMax^®^ Discover Microplate Reader (Promega, Madison, WI, USA). Unspecific cytotoxicity was determined by measuring the percentage of LDH release into the supernatant of treated cells compared to untreated controls. Measurements were performed in triplicate in *n* = 3 independent experiments and mean percentage changes ± SEM as compared to controls are shown.

#### 4.2.6. Western Blot Analysis

Changes in the level of protein expression were assessed through Western blotting. HepG2 and HuH-7 cells were seeded at a density of 1 × 10^6^ cells/well in 100 mm petri dishes and incubated until the desired confluency. Drugs were added at given concentrations for 24 h. Untreated cells served as controls. After washing with PBS, the cells were frozen at −20 °C. Subsequently, radioimmunoprecipitation assay (RIPA) buffer mixed with one cOmplete™ Mini Protease Inhibitor tablet/10 mL (Roche Diagnostics GmbH, Mannheim, Germany) was added to lyse the cells. Following these steps, the protein levels were analyzed using the Pierce™ BCA Protein Assay Kit (Thermo Fisher Scientific, Waltham, USA) and protein levels were normalized to ensure equal protein loading of 20 µg/lane. Laemmli buffer and β-mercaptoethanol were added to the probes following denaturation at 96 °C for 10 min. Proteins were separated using SDS–PAGE and electro-transferred to polyvinylidene difluoride (PVDF) membranes. Membranes were blocked in 5% non-fat dry milk in Tris-buffered saline with 0.1% Tween-20 and incubated with primary antibody overnight at 4 °C. Primary antibodies included Hsp90α (Gentaur, Aachen, Germany, E8ET1605-57, 1:4000), Hsp70 (Santa Cruz Biotechnology, Dallas, TX, USA, sc-66048, 1:2000) and β-actin (Sigma-Aldrich, Munich, Germany, A5441, 1:8000). Secondary peroxidase-coupled anti-IgG anti-mouse (926-80010 LI-COR Biotechnology GmbH, Bad Homburg, Germany, 1:10,000) or anti-rabbit (926-80011 LI-COR Biotechnology GmbH, Bad Homburg, Germany, 1:10,000) antibodies were incubated for 1 h at room temperature. Bands were visualized using Clarity and Clarity Max Western Blotting ECL Substrates (Bio-Rad Laboratories, Munich, Germany) and a ChemiDoc MP Imaging System (Bio-Rad Laboratories, Munich, Germany). Blots of *n* = 3 independent experiments were generated. β-Actin expression served as a loading control and calculated values were normalized to its expression.

### 4.3. Toxoplasma Gondii Testing Assay

Cells of the Vero cell line (ATCC^®^ CCL81™, Manassas, VA, USA) were used to cultivate *T. gondii* tachyzoites (RH strain, a gift from Dr. Saeed El-Ashram, State Key Laboratory for Agrobiotechnology, China Agricultural University, Beijing, China). Effects of test compounds on *T. gondii* growth were investigated as published previously and expressed as IC_50_ values, which were calculated from three independent experiments [63,89].

### 4.4. Leishmania Major Testing Assays

Promastigotes (obtained from a patient in 2016) and amastigotes of *L. major* were applied for drug testing according to previously published methods [63,90]. IC_50_ values were calculated from three independent experiments. Laboratory animal experiments with BALB/c mice (from the Pharmaceutical College, King Saud University, Kingdom of Saudi Arabia) for the generation of parasites and macrophages were performed strictly following the rules and guidelines of the responsible committee of research ethics, Deanship of Scientific Research, Qassim University, Kingdom of Saudi Arabia (permission number 20-03-20).

### 4.5. Vero Cell and Macrophage Cytotoxicity

Cytotoxicity to Vero cells or murine macrophages was evaluated using the MTT assay according to previously published articles and expressed as IC_50_ values which were determined from three independent experiments [63,91].

### 4.6. Statistical Analysis

Visualization of data and statistical calculations were performed using GraphPad Prism 10 (GraphPad Software Version 10.4.0, San Diego, CA USA).

## Figures and Tables

**Table 1:** Antiproliferative activity (expressed as IC_50_ values) of the indicated chalcones against HCC cell lines HepG2 and HuH-7 after 48 h (crystal violet assay).

Compounds	HepG2 [µM]	HuH-7 [µM]
SU086	>10	>10
246TMP-3SF5	1.3 ± 0.2	2.2 ± 0.3
24DMP-3NO	>10	>10
2MeO-Anth	>10	>10
24DMP-Anth	>10	>10
246TMP-Anth	>10	>10
4NO-Fc	5.2 ± 0.8	4.4 ± 0.5
Fc-Anth	>23	>23
17-AAG	1.6 ± 0.5	0.4 ± 0.4
Sorafenib	4.4 ± 1.0	5.4 ± 0.6

**Table 2:** Activities (expressed as IC_50_ values) of chalcones against *T. gondii* parasites and Vero monkey kidney cells. Atovaquone (ATO) was used as a positive control.

Compounds	*T. gondii* [µM]	Vero [µM]	SI ^1^
SU086	3.8	9.0	2.4
246TMP-3SF5	0.42	1.2	2.8
24DMP-3NO	2.2	5.4	2.4
2MeO-Anth	9.8	22.8	2.3
24DMP-Anth	7.6	22.3	2.9
246TMP-Anth	5.8	19.6	3.4
4NO-Fc	18.8	23.0	1.2
Fc-Anth	6.7	19.5	2.9
ATO ^2^	0.07	9.5	135

^1^ SI = selectivity index (Vero/*T. gondii*). ^2^ Values taken from [63].

**Table 3:** Activities (expressed as IC_50_ values) of chalcones against *L. major* parasites (promastigotes and amastigotes) and macrophages. Amphotericin B (AmB) was used as a positive control.

Compounds	Promastigotes [µM]	Amastigotes [µM]	Macrophages [µM]	SI (Promastigotes) ^1^	SI (Amastigotes) ^1^
SU086	0.41	3.5	11.1	27.1	3.2
246TMP-3SF5	9.2	20.3	32.1	3.5	1.6
24DMP-3NO	0.86	28.4	25.2	29.3	0.89
2MeO-Anth	1.8	33.7	48.8	26.6	1.5
24DMP-Anth	2.2	28.0	48.3	22.3	1.7
246TMP-Anth	0.73	18.8	15.8	21.7	0.84
4NO-Fc	6.7	19.9	18.6	2.8	0.93
Fc-Anth	6.7	21.1	20.2	3.0	1.0
AmB ^2^	0.83	0.47	8.1	9.8	17.2

^1^ SI = selectivity index (macrophages/*L. major*). ^2^ Values taken from [63].

## Data Availability

Original data can be obtained from the authors upon reasonable request.

## Supplementary Materials

The following supporting information can be downloaded at: https://www.mdpi.com/article/10.3390/ph18010050/s1, NMR data of known chalcones, Scheme S1: Claisen–Schmidt reaction scheme; Figure S1: ^1^H NMR spectrum of 246TMP-3SF5; Figure S2: ^13^C NMR spectrum of 246TMP-3SF5; Figure S3: HRMS of 246TMP-3SF5; Figure S4: ^1^H NMR spectrum of 246TMP-Anth; Figure S5: HRMS of 246TMP-Anth.
