# Supplementary material for: Identification of a New Pentafluorosulfanyl-Substituted Chalcone with Activity Against Hepatoma and Human Parasites
Source: Pharmaceuticals (Basel). 2025 Jan 3;18(1):50. doi: 10.3390/ph18010050 (PMC11768771; doi:10.3390/ph18010050)

Supplementary Materials

New Pentafluorosulfanyl-substituted Chalcone with Activity Against Hepatoma and Human Parasites

Alessandra Viperino [1], Michael Höpfner [1], Nicole Edel [1], Ibrahim S. Al Nasr [2], Waleed S. Koko [2], Tariq A. Khan [3], Imen Ben Abdelmalek [2], Rainer Schobert [5], Bernhard Biersack [5,*] and Bianca Nitzsche [1,*]

[1] Institute of Physiology, Charité-Universitätsmedizin Berlin, Corporate Member of the Freie Universität Berlin, Humboldt-Universität zu Berlin and Berlin Institute of Health, Charitéplatz 1, 10117 Berlin, Germany; alessandra.viperino@charite.de, michael.hoepfner@charite.de, nicole-edel@t-online.de, bianca.nitzsche@charite.de
[2] Department of Biology, College of Science, Qassim University, Qassim 51452, Saudi Arabia; insar@qu.edu.sa, wasyko2002@yahoo.com, mm.abdulmalek@qu.edu.sa
[3] Department of Basic Health Sciences, College of Applied Medical Sciences, Qassim University, Ar Rass 51921, Saudi Arabia; sirtariqayub@gmail.com
[4] Organic Chemistry Laboratory, University Bayreuth, Universitätsstrasse 30, 95440 Bayreuth, Germany; rainer.schobert@uni-bayreuth.de, bernhard.biersack@yahoo.com

* Correspondence: bernhard.biersack@yahoo.com (B.B.), bianca.nitzsche@charite.de (B.N.),

NMR data of known chalcones SU086, 24DMP-3NO, 2MeO-Anth, 24DMP-Anth, 4NO-Fc, and Fc-Anth

SU086

^{1}H NMR (300 MHz, $CDCl_3$) δ 3.77 (6 H, s), 3.85 (3 H, s), 6.15 (2 H, s), 7.04 (1 H, d, J = 16.0 Hz), 7.41 (1 H, d, J = 16.0 Hz), 7.5-7.6 (1 H, m), 7.8-7.9 (1 H, m), 8.2-8.3 (1 H, m), 8.33 (1 H, s); ^{13}C NMR (75.5 MHz, $CDCl_3$) δ 55.4, 55.9, 90.6, 111.4, 122.7, 124.2, 129.8, 131.4, 133.7, 137.0, 140.1, 148.6, 159.1, 162.9, 193.0.

24DMP-3NO

^{1}H NMR (300 MHz, $CDCl_3$) δ 3.86 (3 H, s), 3.92 (3 H, s), 6.48 (1 H, s), 6.5-6.6 (1 H, m), 7.5-7.6 (1 H, m), 7.65 (2 H, s), 7.8-7.9 (2 H, m), 8.1-8.2 (1 H, m), 8.42 (1 H, s); ^{13}C NMR (75.5 MHz, $CDCl_3$) δ 55.6, 55.8, 98.6, 105.5, 121.5, 122.2, 124.0, 129.8, 133.2, 134.1, 137.4, 138.4, 148.7, 160.7, 164.7, 189.3.

2MeO-Anth

^{1}H NMR (300 MHz, $CDCl_3$) δ 3.92 (3 H, s), 7.0-7.1 (2 H, m), 7.37 (1 H, d, J = 16.1 Hz), 7.4-7.5 (5 H, m), 7.7-7.8 (1 H, m), 8.0-8.1 (2 H, m), 8.3-8.4 (2 H, m), 8.44 (1 H, s), 8.59 (1 H, d, J = 16.1 Hz).

24DMP-Anth

^{1}H NMR (300 MHz, $CDCl_3$) δ 3.87 (3 H, s), 3.88 (3 H, s), 6.48 (1 H, s), 6.5-6.6 (1 H, m), 7.4-7.5 (5 H, m), 7.89 (1 H, d, J = 8.7 Hz), 7.9-8.0 (2 H, m), 8.3-8.5 (2 H, m), 8.61 (1 H, d, J = 16.0 Hz).

4NO-Fc

^{1}H NMR (300 MHz, $CDCl_3$) δ 4.2-4.3 (5 H, m), 4.6-4.7 (2 H, m), 4.9-5.0 (2 H, m), 7.1-7.2 (2 H, m), 7.7-7.8 (2 H, m), 8.2-8.3 (2 H, m).

Fc-Anth

^{1}H NMR (300 MHz, $CDCl_3$) δ 4.2-4.3 (5 H, m), 4.5-4.6 (2 H, m), 4.9-5.0 (2 H, m), 7.11 (1 H, d, J = 15.9 Hz), 7.5-7.6 (4 H, m), 8.0-8.1 (2 H, m), 8.3-8.4 (2 H, m), 8.47 (1 H, s), 8.71 (1 H, d, J = 15.9 Hz); ^{13}C NMR (75.5 MHz, $CDCl_3$) δ 69.9, 70.1, 73.0, 80.5, 125.4, 126.2, 128.0, 129.7, 130.7, 131.4, 137.7, 192.4.

Scheme S1. Reagents and conditions: (i) Aryl aldehyde, aqueous NaOH, EtOH, r.t., 24 h.

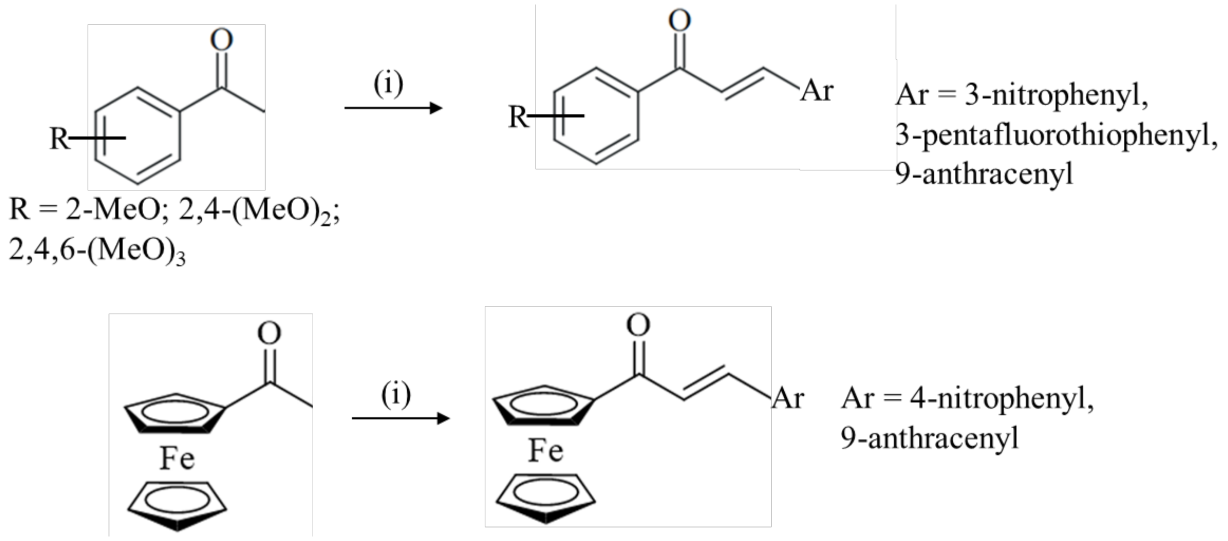

Figure S1. ^{1}H NMR spectrum of 246TMP-3SF5

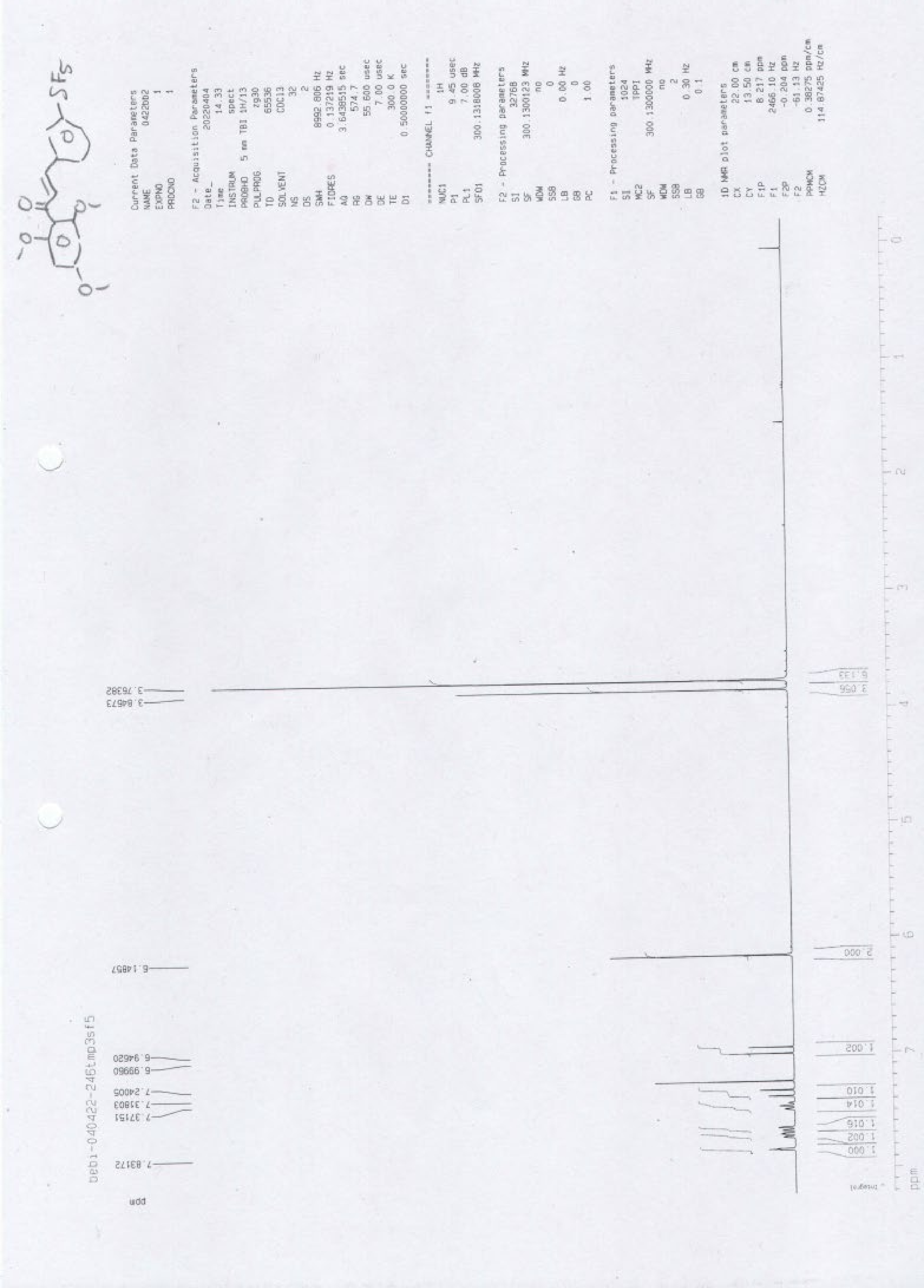

Figure S2. ^{13}C NMR spectrum of 246TMP-3SF5

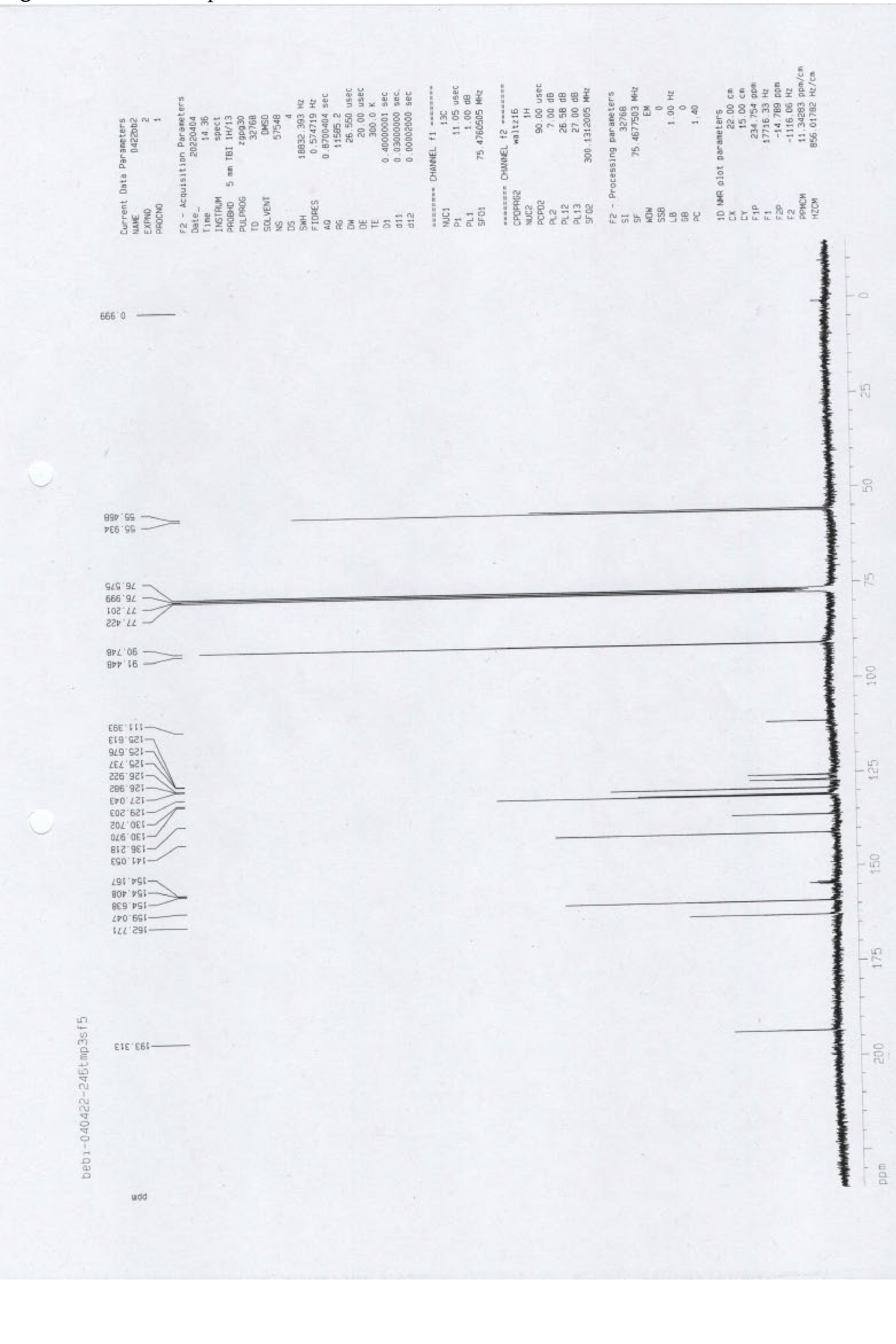

Figure S3. HRMS spectrum of 246TMP-3SF5

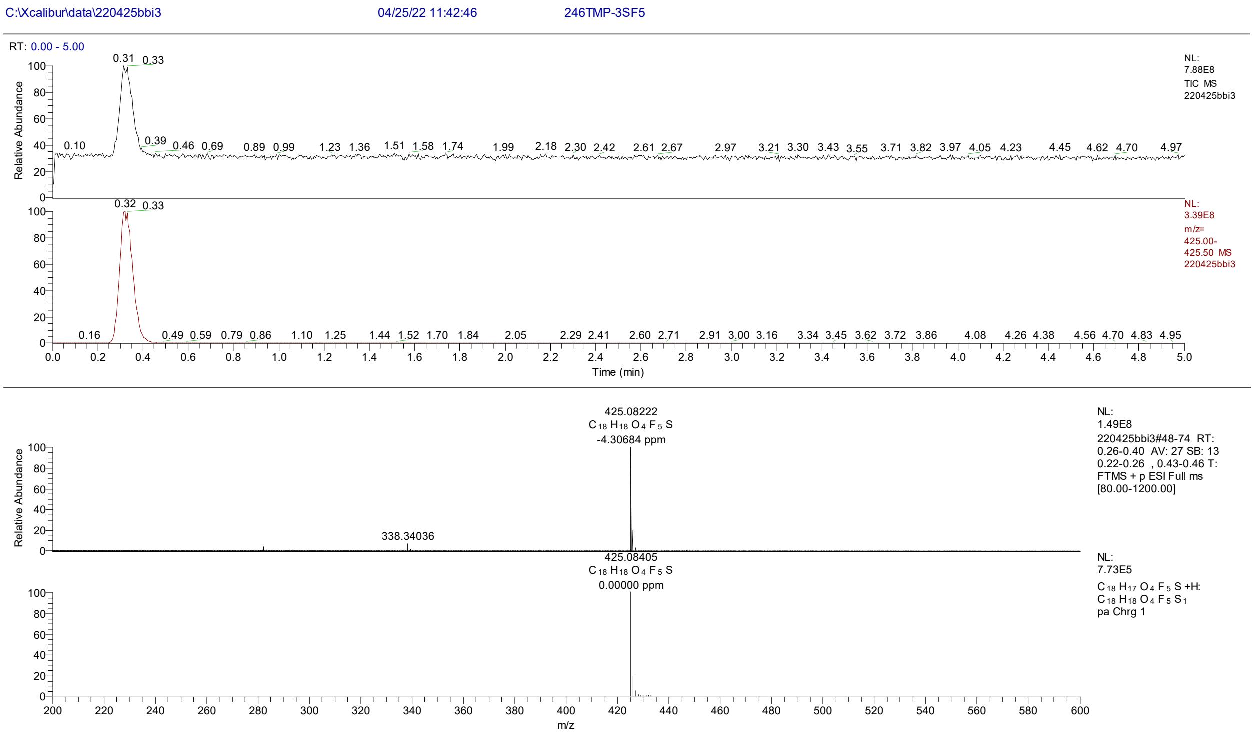

Figure S4. ^{1}H NMR spectrum of 246TMP-Anth

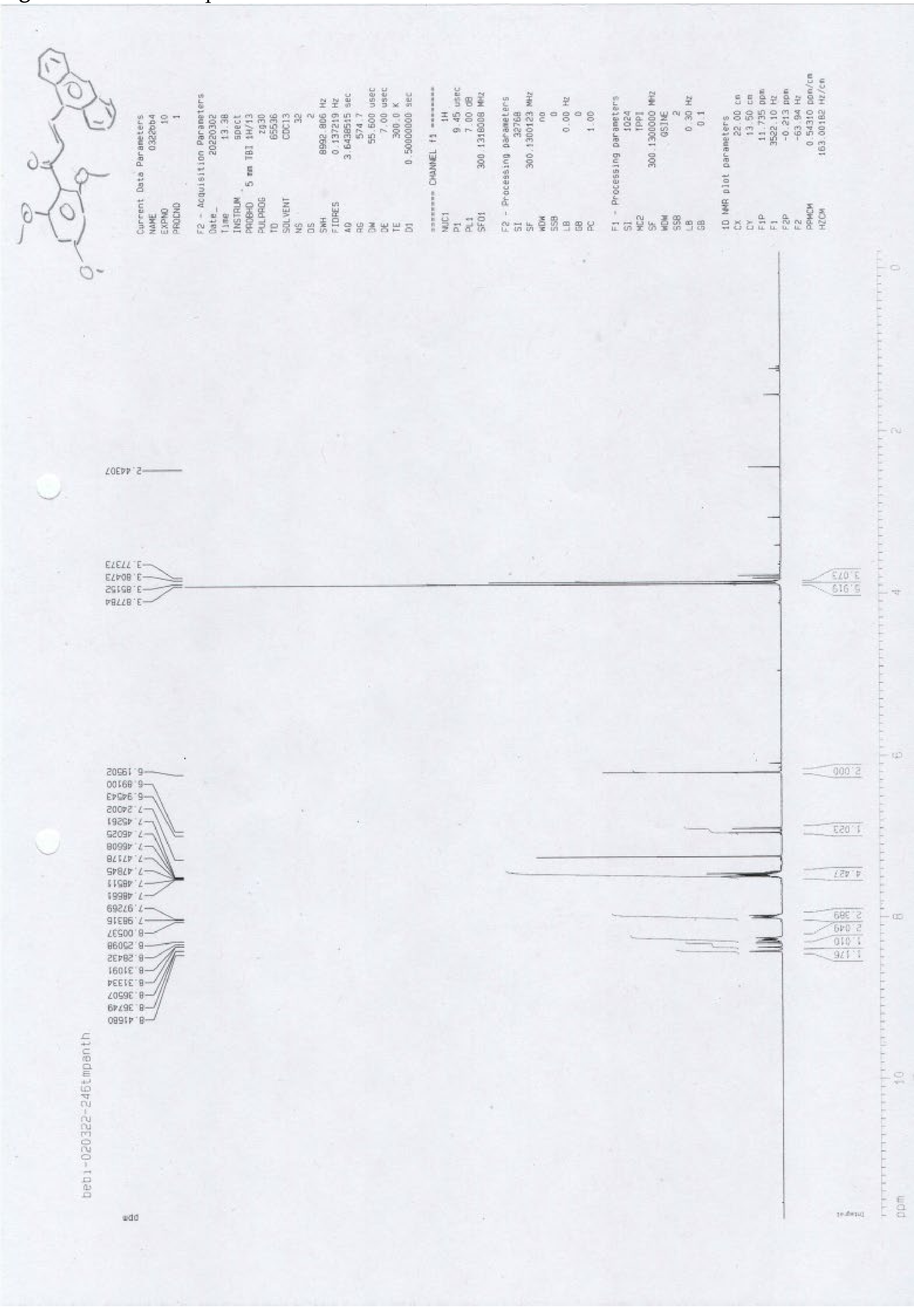

Figure S5. HRMS spectrum of 246TMP-Anth

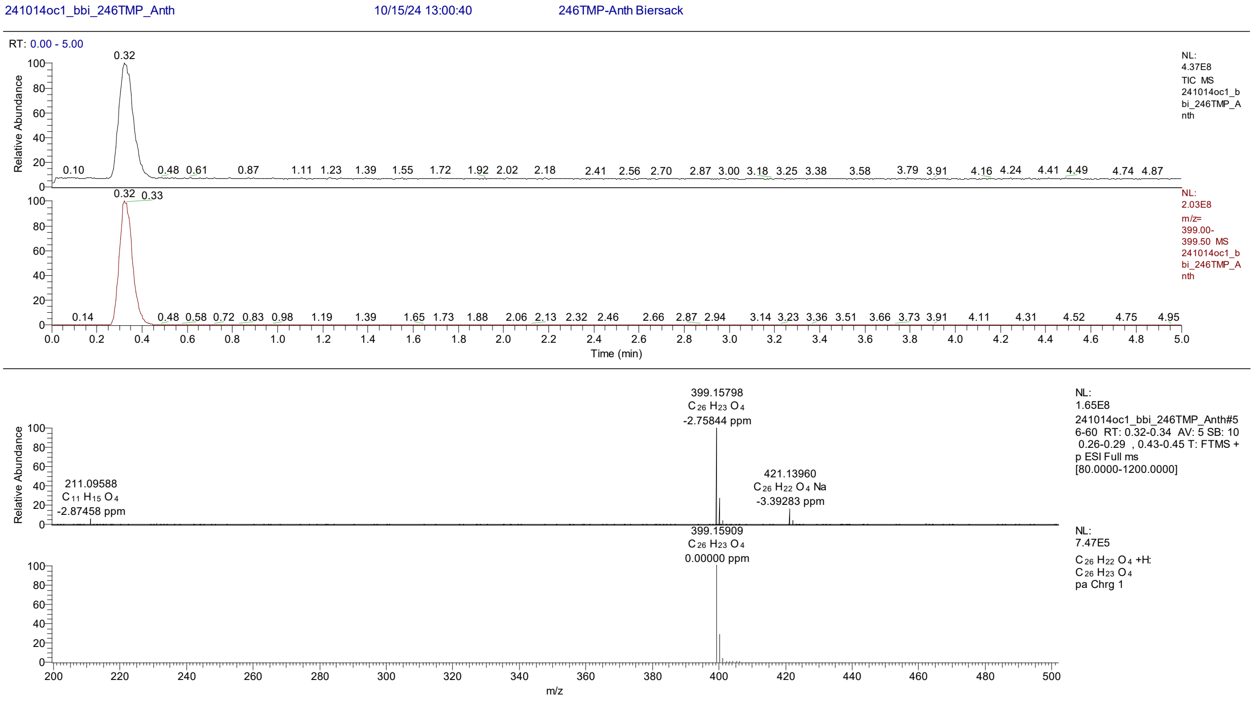

Supplement: Supplementary file 1 [file pharmaceuticals-18-00050-s001.zip › pharmaceuticals-3392394-supplementary.pdf]
